# Forced expiration technique: impact on the respiratory mechanics parameters of children and adolescents with cystic fibrosis

**DOI:** 10.1590/1984-0462/2025/43/2024155

**Published:** 2025-03-24

**Authors:** Patricia Morgana Rentz Keil, Renata Maba Gonçalves Wamosy, Tayná Castilho, Juliana Cardoso, Camila Isabel Santos Schivinski

**Affiliations:** a Universidade de Santa Catarina, Florianópolis, SC, Brazil.; b Universidade de Campinas, Campinas, SP, Brazil.

**Keywords:** Adolescent, Child, Cystic fibrosis, Physical therapy modalities, Respiratory function tests, Adolescente, Criança, Fibrose cística, Fisioterapia respiratória, Testes de função respiratória

## Abstract

**Objective:**

Determine the immediate effect of forced expiration technique (FET) on the respiratory mechanics of children and adolescents with cystic fibrosis (CF). As a secondary objective, the effect of cough induced by FET was evaluated by comparing respiratory mechanics and lung function between those who coughed and those who did not during the FET.

**Methods:**

A before-after clinical trial was conducted with children and adolescents with CF aged six to 15 years. Respiratory mechanics parameters were assessed using the impulse oscillometry system (IOS) in three stages: basal IOS, post-huff IOS, and final post-diaphragmatic breathing exercises (DBE) IOS. For the intervention, FET was requested with five low-volume followed by three high-volume huffs, and finally ten DBE repetitions. Coughing occurred randomly and was not previously requested. To investigate whether FET-induced coughing alters oscillometric parameters, the participants were divided into two groups: those who presented with cough (CG) during the protocol and those who did not (NCG).

**Results:**

Forty-three children and adolescents with CF participated in the study (51.2% female), with an average age of 10.44±2.64 years, where forced expiratory value — FEV_1_=78.51±23.28%, and body mass index — BMI=17.18±2.24 kg/m^2^. The huffing sequence increased all oscillometric parameters, while DBE repetitions led to an increase in these parameters, without a complete return to baseline values. In terms of coughing, there was no significant difference between the NCG and CG in any of the parameters studied.

**Conclusions:**

It was observed that, during the FET, diaphragmatic breathing exercises can attenuate the effort exerted by the forced expiratory maneuver on the airways.

## RESUMO

**Objetivo:**

Investigar o efeito imediato da técnica de expiração forçada (TEF) na mecânica respiratória de crianças e adolescentes com fibrose cística (FC). Como objetivo secundário, avaliar o efeito da tosse induzida pela TEF, comparando a mecânica respiratória e a função pulmonar entre aqueles que tossiram e aqueles que não tossiram durante a TEF.

**Métodos:**

Ensaio clínico de caráter *before-after*, realizado com crianças e adolescentes com FC de seis a 15 anos. Avaliação dos parâmetros da mecânica respiratória, por meio do sistema de oscilometria de impulso (IOS), em três etapas: IOS basal, IOS pós-*huffs* e IOS final pós-exercício de respiração diafragmática (ERD). Para a intervenção, solicitou-se a TEF com: cinco *huffs* a baixo volume, seguidos de três *huffs* a alto volume, e por fim dez repetições do ERD. A tosse ocorreu ao acaso, e não previamente solicitada. Para investigar se a sua ocorrência altera os parâmetros oscilométricos, os participantes foram divididos em dois grupos: os que apresentaram tosse (GCT) e os que não apresentaram durante o protocolo (GST).

**Resultados:**

Participaram 43 crianças e adolescentes com FC (51,2% sexo feminino), com média de idade de 10,44±2,64 anos, valor expiratório forçado — VEF_1_: 78,51±23,28% e índice de massa corporal — IMC de 17,18±2,24 kg/m^2^. A sequência de *huffing* aumentou todos os parâmetros oscilométricos, enquanto as repetições de ERD promoveram aumento desses parâmetros, sem retorno completo aos valores basais. Considerando-se a tosse, não houve diferença significativa entre GST e GCT em nenhum dos parâmetros estudados.

**Conclusões:**

Verificou-se que, durante a TEF, os ERD podem atenuar o esforço exercido pela manobra expiratória forçada sobre as vias aéreas.

## INTRODUCTION

Respiratory physiotherapy is considered important in managing individuals with cystic fibrosis (CF) due to the progressive impairment of the airways resulting from chronic respiratory infections and repeated cycles of inflammation. It is a therapeutic method indicated as part of patients’ daily routine, starting shortly after disease diagnosis, due to its benefits in helping displace viscous and difficult-to-clear mucus.^1,2^

There are several instrumental resources and physiotherapeutic techniques aimed at airway clearance, and the choice of the most suitable intervention should be based on individual age, preferences, and clinical condition.^2^ Among the available technique, the forced expiration technique (FET) stands out because it combines forced expirations or huffs, performed at different lung volumes, with periods of relaxation through respiratory control, also known as the diaphragmatic breathing exercise (DBE).^3,4^

Bronchial clearance techniques lead to coughing, which is a physiological mechanism for protecting the airways. Coughing during FET may occur spontaneously in some individuals. However, the impact of coughing on airway stability warrants further investigation, given its potential to cause airway narrowing.^5,6^

Some studies have assessed the effect of FET in CF patients, either applied alone or in combination with other therapeutic interventions such as postural drainage, high-frequency oscillation, thoracic percussion, and the active cycle of breathing techniques.^3,7,8^ These studies found that FET helped increase expectoration,^3^ improve cardiopulmonary parameters,^8^ and enhance spirometric measures of lung function.^7^

Pulmonary function analysis is commonly used to assess therapeutic responses in cystic fibrosis, as spirometric parameters are associated with disease progression.^9^ Another method, impulse oscillometry (IOS), allows for the evaluation of respiratory mechanics, such as tidal volume, without requiring forced expiratory maneuvers, making it well-accepted in pediatrics.^10^ IOS is also considered a sensitive tool for detecting early airway changes and is used to monitor the progression of pulmonary deterioration during acute exacerbations and the response after treatment.^11^

In patients with CF, there is a recurring need to perform bronchial clearance maneuvers aimed at reducing the risk of infections and respiratory complications. Research demonstrates the benefits of the FET, which promotes controlled expirations and facilitates the clearance and removal of mucus. This technique is widely utilized in clinical practice and is recommended for independent implementation in the daily routine of patients with CF. However, a comprehensive understanding of the immediate effects of FET on respiratory mechanics, as well as the necessity for detailed protocols, requires further investigation.^3,7,8^

Given the above and the need to demonstrate the effect of FET in pediatric patients with CF using referenced instruments for respiratory assessment, the present study aimed to determine the immediate effect of FET on the respiratory mechanics of children and adolescents with CF. As a secondary objective, the effect of cough induced by FET was evaluated by comparing respiratory mechanics and lung function between those who coughed and those who did not during the execution of the technique.

## METHOD

This was a before-after clinical trial conducted at the Joana de Gusmão Children’s Hospital (HIJG) in Florianópolis, Santa Catarina state, Brazil, approved by the Research Ethics Committee (Certificate of Presentation for Ethical Appreciation 80800217.4.0000.5361) and registered in the Brazilian Clinical Trials Registry (REBEC) under number RBR-2zsbgd. Schoolchildren aged six to 15 years with confirmed CF, according to Brazilian guidelines, participated in the study.^1^

Individuals were selected using non-probabilistic convenience sampling, and eligible participants were under follow-up at the referral center, collaborative, and clinically stable based on specific scores. All participants and their parents/guardians were invited and informed about the study, the latter giving their written informed consent and the children and adolescents agreeing to participate. Individuals who did not adequately perform any assessment, compromising the data obtained, were excluded.

Individual data collection occurred on a single day during the outpatient clinic routine at the referral center. Initially, two clinical scores were applied to assess the clinical stability of the participant: the Cystic Fibrosis Clinical Score (CFCS) and the Cystic Fibrosis Foundation Score (CFFS).^12,13^ The CFCS includes five common symptoms and five physical findings, with a final score of 25 or higher indicating pulmonary exacerbation,^12^ as well as the presence of four or more signs and symptoms in the CFFS.^13^

After applying the scores, clinical data were recorded. To that end, the medical records were consulted, considering data on pathogen colonization (presence or absence of bacterial colonization), genetic mutation (presence of one or two ∆F508 alleles, or another mutation), and the spirometric parameters of forced vital capacity (FVC), force expiratory value (FEV_1_), forced expiratory flow between 25 and 75% of FVC (FEF25–75%), and peak expiratory flow (PEF), recorded in absolute and percentage of predicted values according to Polgar^14^ and Knudson et al.,^15^ as well as z-score, lower limit of normal (LLN), and percentage of predicted values according to the Global Lung Function Initiative (GLI).^16^ Disease severity was classified by FEV_1_ values according to Marshall et al.,^17^ with FEV_1_ >70% classified as mild, FEV_1_ >40–69% moderate, and FEV_1_ <40% severe. The spirometric parameters utilized in this study were collected during the outpatient routine by the HIJG laboratory team on the same day and following the execution of the present protocol, thereby providing an up-to-date assessment of the pulmonary function of the studied population. Disease severity was also classified according to the Schwachman-Doershuk score (SDS),^18^ applied and updated by the medical team during routine outpatient consultations. Data collected in the last three months before the protocol were considered for registration. Next, the body mass index (BMI) was calculated and classified through the Ministry of Health’s Telessaúde program (Virtual Health Library, 2018), based on body weight (in kg — Wiso digital glass scale, model Ultra Slim W903) and height (m) measured by the research team using a Sanny^®^ portable stadiometer.

Forced expiration follows the physiological mechanism of the displacement of the equal pressure point (EPP). At EPP, the pressures in and outside the airways are equal, with consequent dynamic compression of the bronchial pathway towards the mouth, causing shearing and changes in mucus rheology, favoring mucus displacement and consequent expectoration.^5^ The last stage of FET consists of a period of respiratory control through DBE. In DBE, the individual performs inspiration and expiration with greater movement of the abdominal wall and less of the rib cage, aiming to maintain airway stability, prevent bronchospasm, and prevent a decreased peripheral oxygen saturation.^3,4^

The 5-step intervention protocol was then conducted, as follows:

1. Baseline IOS — three IOS maneuvers were recorded for 20 seconds each;
2. The child performed five low-volume followed by three high-volume huffs;
3. An IOS maneuver immediately after the huff sequence;
4. Ten DBE repetitions; and
5. A final IOS maneuver.

**Figure 1:**
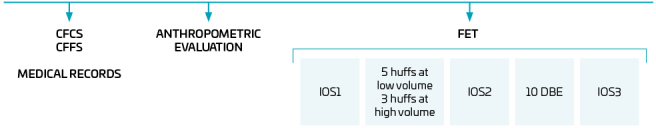
Flowchart of the assessment procedures and respiratory mechanics parameters obtained during the intervention protocol.

CFCS: Cystic Fibrosis Clinical Score; CFFS: Cystic Fibrosis Foundation Score; IOS: Impulse Oscillometry System; FET: forced expiration technique; DBE: Diaphragmatic Breathing Exercise; Medical Records: genetic mutation consultation, colonization data, spirometry, and most recent sputum culture results; IOS1: three basal IOS maneuvers; IOS2: one post-huff IOS maneuver; IOS3: one post-DBE IOS maneuver.

Steps 3 and 5 were conducted to determine the immediate effect of the isolated huff (3) and FET itself (5), which includes DBE (Figure 1). For baseline IOS (1), three maneuvers lasting 20 seconds each were recorded, with parameter values not varying more than 10%.^19^ The maneuver with the lowest R5 and R20 values was considered for analysis. For IOS 3) and 5), a single IOS maneuver was performed to ensure the recording of the immediate effect of the huff sequence and DBE, respectively.

The entire intervention protocol was carried out with the child seated, feet flat on the floor, and head in a neutral position. The researcher demonstrated the technique, where huffs at different lung volumes were performed, followed by DBE. Always preceded by inspirations, low- and high-volume huffs were directed to clear peripheral and proximal airways, respectively.^5^ Participants were familiar with the techniques since they received guidance and training during routine consultations at the referral center. After the last huff sequence and an IOS maneuver, ten DBE were conducted. For DBE, the schoolchildren were verbally instructed on inspiration and expiration, based on literature studies with adults.^20,21^ Given that it was a pediatric population, only the verbal command varied according to the age group during the DBE. Children aged six to ten years were asked to “breathe in slowly through your nose, filling your belly with air” and “breathe out slowly through your mouth, sucking in the belly.” Children older than ten years old were instructed to “perform diaphragmatic breathing calmly.” During DBE, the researcher applied manual proprioceptive stimulation on the abdomen and upper chest.^21^

All maneuvers were conducted by the same physiotherapist, who asked that coughing be avoided throughout the protocol. In cases where coughing occurred, the protocol was followed, the event recorded, and the children were allocated to the cough group (CG) for later comparison with their non-cough (NCG) counterparts. Given the importance of evaluating the effect of coughing on the airways after FET application, since coughing is a physiological mechanism for airway clearance and may potentially cause airway narrowing, dividing the groups aims to investigate whether the presence of FET-induced coughing alters the oscillometric parameters assessed.

Respiratory mechanics parameters were assessed using the Master Screen IOS pneumotachograph (Erich Jaeger, Germany^®^), according to the American Thoracic Society (ATS) recommendations.^22^ The following oscillometric parameters were analyzed: impedance at 5 Hz (Z5), total airway resistance (R5), central airway resistance (R20), and reactance at 5 Hz (X5), expressed in absolute and percentage of predicted values, according to Assumpção et al.^23^

IBM Statistical Package for the Social Sciences (SPSS) software version 20.0 was used for statistical and descriptive data analysis. Data distribution was assessed using the Shapiro-Wilk test, and the *t* test or Mann-Whitney test for sample characterization and comparison of spirometric data between the NCG and CG. Oscillometric results for the whole sample and between the NC and CG were analyzed by two-way repeated measures analysis of variance (ANOVA), with a double effect (group and intervention), and the Bonferroni post-hoc test. A 5% significance level was adopted for all tests.

The sample size was calculated using the online calculator at (http://powerandsamplesize.com/Calculators/Compare-k-Means/1-Way-ANOVA-Pairwise) based on the R5 parameter data obtained in a pilot study with 12 individuals, six in each group (NCG and CG). For an R5 mean of 0.5 and 0.6 kPa/L/s, with a standard deviation of 0.13 kPa/L/s, a test power of 0.85, and a type I error rate of 5%, it was estimated that 17 individuals in each group were sufficient.

## RESULTS

A total of 46 schoolchildren with CF participated in the study, three of whom were excluded for not adequately performing the IOS. The final sample consisted of 43 subjects, 25 allocated to the NCG group and 18 to the CG. No significant differences in age and anthropometric characteristics were observed between the two groups (Table 1).

**Table 1:** Age and anthropometric variables, expressed as mean, standard deviation, median and confidence interval of the total sample (cough group and non-cough group).

Variable	Total Group (n=43) M±SD (95%CI)	NCG (n=25) M±SD (95%CI)	CG (n=18) M±SD (95%CI)	p-value
Age (years)	10.44±2.64 (9.63–11.25)	10.36±2.81 (9.20–11.52)	10.56±2.46 (9.33–11.78)	0.840*
Female (%)	51.2	44.0	61.1	-
Weight (kg)	35.71±11.30 (32.23–39.19)	36.06±11.48 (31.32–40.80)	35.22±11.36 (29.57–40.87)	0.814*
Height (cm)	142.16±16.00 (137.24–147.09)	142.32±16.48 (135.52–149.12)	141.94±15.77 (134.10–149.79)	0.840*
BMI (kg/m2)	17.18±2.24 (16.49–17.87)	17.33±2.22 (16.41–18.24)	16.98±2.31 (15.84–18.13)	0.630^†^
Normal weight (%)	86.05	88	83.33	-
SDS classification (%)				
Excellent	46.5	60	27.8	-
Good	46.5	32	66.7	-
Average	7	8	5.5	-
Poor	0	0	0	-
Mutation classification (%)				
F508del heterozygous	48.8	36.0	66.7	-
F508del homozygous	30.2	40	16.7	-
Others	20.9	24	16.6	-
Pathogen colonization (%)	62.8	60	66.7	-

M: mean; SD: standard deviation; 95%CI: 95% confidence interval for the mean; n: number; kg: kilogram; cm: centimeter; BMI: body mass index; kg/m2: kilogram per square meter; NCG: non-cough group; CG: cough group; SDS: Schwachman-Doershuk score; p-value: comparison between NCG and CG by the t and Mann-Whitney tests.

*p≤0.05, according to the Mann-Whitney U test

^†^p≤0.05, according to the independent t-test.

Spirometric data of the total sample are presented in Table 2, which shows a mean FEV1 of 78.51±23.28%, classified as mild severity.^17^ There was no difference between the NCG and GC regarding lung function.

**Table 2:** Spirometric data, expressed as mean, standard deviation, median and confidence interval of the total sample (non-cough group and cough group), as well as a comparison between them, according to the t-test or Mann-Whitney test.

Spirometric parameters	Total Group (n=43) M±SD (95%CI)	NCG (n=25) M±SD (95%CI)	CG (n=18) M±SD (95%CI)	p-value
FVC (L)	2.26±0.71 (2.05–2.48)	2.37±0.72 (2.07–2.66)	2.13±0.68 (1.78–2.47)	0.280*
FVC (%)	93.59±20.50 (87.28–99.90)	96.80±19.79 (88.63–104.97)	89.14±21.21 (78.59–99.68)	0.231*
FVC (LLN)	2.01±0.67 (1.80–2.21)	2.02±0.71 (1.73–2.32)	1.99±0.62 (1.68–2.30)	0.863^†^
FVC (Z-score)	-0.58±1.80 (-1.13–(-0.03))	-0.26±1.76 (-0.98–0.47)	-1.03±1.80 (-1.93–(-0.13))	0.170*
%GLI FVC	93.57± 21.16 (87.05–100.08)	97.33±20.91 (88.70–105.96)	88.35±20.97 (77.92–98.78)	0.173*
FEV_1_ (L)	1.74±0.62 (1.55–1.93)	1.83±0.58 (1.59–2.07)	1.63±0.66 (1.30–1.96)	0.300*
FEV_1_ (%)	78.51±23.28 (71.34–85.67)	82.14±22.35 (72.91–91.37)	73.46±24.22 (61.41–85.50)	0.232*
FEV_1_ (LLN)	1.76±0.57 (1.58–1.93)	1.77±0.61 (1.52–2.02)	1.74±0.53 (1.48–2.00)	0.863^†^
FEV_1_ (Z-score)	-1.45±1.94 (-2.05–(-0.85))	-1.12±1.89 (-1.90–(-0.34))	-1.90±1.98 (-2.88–(-0.92))	0.200*
%GLI FEV_1_	82.42±23.87 (75.08–89.77)	86.51±23.09 (76.98–96.04)	76.74±24.42 (64.60–88.88)	0.190*
PEF (L)	3.66±1.53 (3.19–4.14)	3.95±1.54 (3.32–4.59)	3.26±1.47 (2.53–3.99)	0.150*
PEF (%)	69.34±22.42 (62.44–76.24)	74.70±20.83 (66.11–83.30)	61.88±22.98 (50.45–73.31)	0.064*
FEF 25–75 (L)	1.67±0.84 (1.41–1.93)	1.74±0.75 (1.43–2.05)	1.58±0.96 (1.10–2.05)	0.530*
FEF 25–75 (%)	60.96±27.08 (52.63–69.30)	63.79±25.52 (53.25–74.32)	57.04±29.40 (42.42–71.66)	0.430*
FEF 25–75% (LLN)	1.72±0.53 (1.56–1.89)	1.71±0.57 (1.48–1.95)	1.74±0.50 (1.49–1.99)	0.941^†^
FEF 25–75% (Z-score)	-1.74±1.50 (-2.20–(-1.28) )	-1.55±1.43 (-2.14–(-0.97))	-2.00±1.60 (-2.79–(-1.20))	0.350*
%GLI FEF25-75%	65.25±28.88 (56.36–74.14)	69.04±27.71 (57.61–80.48)	59.98±30.44 (44.84–75.12)	0.320*

M: mean; SD: standard deviation; n: number; 95%CI: 95% confidence interval for the mean; NCG: non-cough group; CG: cough group; p-value: comparison between NCG and CG; FVC: forced vital capacity; FEV1: forced expiratory volume in one second; PEF: peak expiratory flow; FEF25–75%: forced expiratory flow between 25 and 75% of FVC; GLI: Global Lung Function Initiative; %: percentage of predicted; L: liters; LLN: lower limit of normal; p-value: comparison between the NCG and CG by the t test and Mann-Whitney test.

*p≤0.05 according to the independent t test

^†^p≤0.05 according to the Mann-Whitney U test.

Concerning the main outcome, the comparison between interventions (pre-FET, post-huff, and post-FET) in the whole sample showed a difference in all oscillometric parameters (Z5, R5, R20, and X5), absolute and percentage of predicted values. This difference was observed in the post-hoc analysis between pre- and post-huff stages (p<0.05) and pre- and post-FET (p<0.05) for all parameters (Table 3). In relation to cough, there was no significant difference in any of the parameters studied in the NCG and CG, as shown in Table 4.

**Table 3:** Oscillometric parameters of the total sample, during the forced expiratory technique intervention, and comparison of the results during the protocol.

Oscillometric parameters	Pre-FET M±SD (95%CI)	Post-huff M±DS (95%CI)	Post-FET M±SD (95%CI)	p-value
Z5 (kPa/L/s)	0.68±0.26 (0.60–0.76)	0.80±0.32 (0.70–0.90)	0.79±0.29 (0.70–0.88)	<0.001*^,†,‡^
Z5%	164.79±66.83 (144.22–185.35)	197.04±91.44 (168.90–225.18)	192.73±81.45 (167.67–217.80)	<0.001*^,†,‡^
R5 (kPa/L/s)	0.63±0.23 (0.55–0.70)	0.74±0.28 (0.65–0.82)	0.73±0.26 (0.65–0.81)	<0.001*^,†,‡^
R5%	104.11±27.93 (95.51–112.71)	124.45±37.31 (112.97–135.93)	122.76±37.61 (111.18–134.33)	<0.001*^,†,‡^
R20 (kPa/L/s)	0.47±0.11 (0.44–0.50)	0.53±0.13 (0.49–0.57)	0.52±0.13 (0.48–0.57)	<0.001*^,†,‡^
R20%	95.72±16.74 (90.57–100.87)	109.41±23.95 (102.04–116.78)	107.09±24.25 (99.62–114.55)	<0.001*^,†,‡^
X5 (kPa/L/s)	-0.26±0.14 (-0.31–(-0.22))	-0.30±0.18 (-0.35–(-0.24))	-0.29±0.15 (-0.33–(-0.24))	0.002*^,†,‡^
X5%	191.71±85.27 (165.47–217.95)	214.74±114.22 (179.59–249.89)	207.61±97.24 (177.68–237.53)	0.005*^,†,‡^

M: mean; SD: standard deviation; n: number; 95%CI: 95% confidence interval for the mean; FET: forced expiratory technique; R5: resistance at 5 Hz; R20: resistance at 20 Hz; X5: reactance at 5 Hz; Z5: impedance at 5 Hz (all expressed as kPa/L/s); p-value: intervention effect by the repeated measures ANOVA test with significance at p≤0.05.

*statistically significant differences in the comparison between the intervention effect pre-FET versus post-huff by the repeated measures ANOVA test (Bonferroni post-hoc) with significance at p≤0.05;

^†^statistically significant differences in the comparison between the intervention effect post-huff versus post-FET by the repeated measures ANOVA test (Bonferroni post-hoc) with significance at p≤0.05;

^‡^statistically significant differences in the comparison between the intervention effect pre-FET versus post-FET by the repeated measures ANOVA test (Bonferroni post-hoc) with significance at p≤0.05.

**Table 4:** Distribution of oscillometric parameter data from non-cough group and cough group during forced expiratory technique, and comparison of the data during the application of the technique.

Oscillometric parameters	Pre-FET		Post-huff		Post-FET		p-value
	NCG (n=25) M±SD (95%CI)	CG (n=18) M±SD (95%CI)	NCG (n=25) M±SD (95%CI)	CG (n=18) M±SD (95%CI)	NCG (n=25) M±SD (95%CI)	CG (n=18) M±SD (95%CI)	
Z5 (kPa/L/s)	0.64±0.24 (0.55–0.74]	0.74±0.30 (0.59–0.88)	0.73±0.26 (0.62–0.83)	0.91±0.37 (0.72–1.09)	0.74±0.27 (0.63–0.86)	0.84±0.31 (0.69–1.00)	0.216
Z5%	162.88±70.45 (133.81–191.96]	167.43±63.37 (135.91–198.94)	190.08±101.03 (148.38–231.78)	206.71±77.94 (167.95–245.47)	190.26±84.96 (155.19–225.33)	196.16±78.59 (157.08–235.24)	0.095
R5 (kPa/L/s)	0.59±0.21 (0.51–0.68]	0.67±0.26 (0.54–0.80)	0.68±0.23 (0.58–0.77)	0.83±0.32 (0.67–0.99)	0.70±0.25 (0.59–0.80)	0.77±0.27 (0.64–0.91)	0.250
R5%	99.62±24.33 (89.57–109.66]	110.36±31.95 (94.47–126.25)	115.14±31.47 (102.14–128.13)	137.39±41.66 (116.67–158.11)	117.44±32.26 (104.12–130.76)	130.14±43.87 (108.32–151.96)	0.702
R20 (kPa/L/s)	0.46±0.11 (0.42–0.50]	0.48±0.10 (0.43–0.54)	0.51±0.13 (0.46–0.57)	0.56±0.13 (0.50–0.63)	0.52±0.14 (0.46–0.57)	0.54±0.13 (0.47–0.60)	0.539
R20%	92.78±15.47 (86.39–99.16]	99.81±18.00 (90.86–108.76)	104.49±22.04 (95.40–113.59)	116.24±25.43 (103.60–128.89)	104.21±21.64 (95.28–113.14)	111.08±27.61 (97.35–124.81)	0.156
X5 (kPa/L/s)	-0.24±0.13 (-0.29–(-0.19)]	-0.30±0.15 (-0.37–(-0.22))	-0.25±0.15 (-0.32–(-0.19))	-0.35±0.20 (-0.45–(-0.26))	-0.26±0.14 (-0.31–(-0.20))	-0.33±0.17 (-0.41–(-0.25))	0.058
X5%	179.71±79.76 (146.79–212.64)	208.37±92.08 (162.58–254.16)	187.74±90.13 (150.54–224.94)	252.24±134.91 (185.15–319.33)	187.85±80.36 (154.68–221.01)	235.05±113.46 (178.63–291.47)	0.118

M: mean; SD: standard deviation; n: number; NCG: non-cough group; CG: cough group; R5: resistance at 5 Hz; R20: resistance at 20 Hz; X5: reactance at 5 Hz; Z5: impedance at 5 Hz (all expressed as kPa/L/s); FET: forced expiration technique; p-value: comparison between the effect of the groups (NCG and CG) by the repeated measures ANOVA test, with a p-value≤0.05.

## DISCUSSION

This study is pioneering in the use of IOS to investigate the immediate effect of FET on the respiratory mechanics of children and adolescents with CF. The IOS emerges as a promising alternative for evaluating the effects of various physiotherapeutic interventions on respiratory mechanics. It is characterized as a non-invasive method that utilizes tidal volume in its measurements.^6,24^ Additionally, this instrument is capable of identifying and differentiating pulmonary impairment associated with the presence of obstructions in the peripheral and central airways, which are the main targets during secretion clearance in FET.^3,10,11^

The present study stands out for evaluating the impact of the FET on respiratory mechanics, monitoring the moments preceding the FET, during its execution, and immediately afterward in children and adolescents with CF. This technique is widely recommended by physiotherapy consensus for CF, and its use encourages patient independence, aiming for its incorporation into the daily treatment routine and promoting autonomy in the bronchial clearance of these patients.^1,25^

With respect to the results in the whole sample, significant changes in IOS parameters were observed during the intervention protocol. The huffing sequence resulted in an increase in all oscillometric parameters when comparing pre- and post-maneuver values (pre- versus post-huff). Subsequently, following the repetitions of DBE, a decrease in these oscillometric parameters was observed (post-huff versus post-FET); however, there was no complete return to baseline values. The increase in these IOS parameters, specifically R5 and R20, which are related to airway resistance, may be due to airway instability in some individuals with CF, with a consequent risk of bronchospasm after forced expirations, as occurs in huffing.^26^

This is because CF is a disease characterized by chronic inflammatory processes and infections resulting from pathogen colonization, which trigger airway remodeling and consequent impairment in the structure and mechanics of the respiratory system. Pulmonary disease progression also determines an imbalance in the ventilation/perfusion ratio, obstruction and increased airway resistance, and decreased lung function.^27^

Another relevant factor is the excessive secretion nature of CF, which promotes the accumulation of secretions in different pulmonary regions.^1^ Due to the different lung volumes mobilized during huffing,^3,4,8,27^ there is a displacement of secretions, which may have resulted in increased airway obstruction and, consequently, an elevation of the oscillometric parameters.

Other studies have also investigated huffing in different health conditions. In CF, Pryor et al.^3^ assessed young adults hospitalized for acute pulmonary exacerbations (PExs) over four days. The study involved various combined physiotherapy techniques, including postural drainage, respiratory expansion exercises, coughing, chest percussion, vibration, FET, and chest compression, applied either by the physiotherapist or the patient themselves. During FET, huffs were performed at medium and low lung volumes, followed by a period of DBE. The authors recommend low lung volume huffing, as it is a less exhausting maneuver compared to a sequence of coughs.

Similarly, Santos et al.^8^ assessed the immediate effects of FET in 18 schoolchildren with CF during hospitalization and discharge for acute PExs. There was an improvement in oxygen saturation, respiratory and heart rate immediately after FET, only upon hospital admission. According to the authors, this improvement was due to the significant obstructive nature that patients exhibit at the beginning of acute PExs, in addition to the airway clearance effect of FET.

Unlike CF, Hasani et al.^28^ analyzed 19 adults with chronic obstructive disease and bronchiectasis who underwent three interventions: respiratory control, cough, and FET. Six huffs per minute were performed for five minutes, with a one-minute rest period after each huff sequence. During respiratory control, the patient only rested. The results showed that coughing and forced expirations were equally effective in bronchial clearance, as demonstrated by the wet weight of sputum collected over the three assessment days. However, coughing demanded more effort from patients, requiring a higher maximum expiratory flow than huffing for the same amount of expectorated secretion. Pontifex et al.^29^ compared the difference in energy expenditure between periods of huffing and directed voluntary coughing in asymptomatic, non-smoking young individuals, finding that it was similar between both and greater when compared to the rest period.

As far as is known, coughing is a defense reflex of the airways that promotes the protection and removal of pulmonary secretions.^30^ This maneuver occurs through dynamic compression in more proximal airways, and in the case of patients prone to instability, collapse may occur.^25^ However, no significant intergroup differences were found when the individuals in the present study were divided into the NCG and CG and their data were compared during the protocol. This lack of impact of coughing on respiratory mechanics in this group suggests several possibilities. One is that the CG had predicted PEF percentages tending toward greater severity (p=0.06) compared to the NCG, indicating greater airway obstruction, which may explain their coughing during the protocol. Greater severity in this group can also be observed by analyzing the absolute baseline values of the X5 parameter, which showed a tendency to be worse (p=0.058) than in the NCG. The X5 parameter represents the elastic recoil properties of peripheral lung tissue, with more negative values in diseases with bronchial obstruction.^24^ In CF, infections and the resulting inflammatory processes occur early in life, leading to changes that affect the peripheral airways and result in a progressive decline in lung function.^31^ The behavior of the X5 parameter in the CG suggests a greater peripheral compromise in participants from this group, with more negative baseline values when compared to the NCG.

Contrary to the information in the literature, which highlights the importance of discouraging coughing during certain respiratory physiotherapy techniques due to the associated energy expenditure and possible dynamic airway compression, and, as a consequence, the general recommendation that coughing be restricted to the end of some procedures,^6,26^ our study did not show an increase in resistance following cough events. There was no worsening of the outcomes assessed in the cough group compared to their non-cough counterparts, which warrants further investigation.

Another point to be highlighted is that spirometry was performed after the protocol to prevent the forced and successive maneuvers of the test from interfering with the study. In contrast, the FET, although it includes the huff — a maneuver also characterized as forced but with an open glottis to facilitate secretion removal—also incorporates diaphragmatic breathing, which alleviates the effort of the maneuver on airway instability and serves a therapeutic purpose.^8,9,26^

The current study observed a reduction in the values of the oscillometric parameters Z5, R5, R20, and X5 after the completion of ten repetitions of DBE, although these values did not fully return to baseline levels. This reduction may be attributed to the respiratory control inherent in the technique, which aims to maintain airway stability, preventing airflow obstruction and a drop in oxygen saturation.^3,4^ This mechanism can be justified by the fact that DBE involves quiet breathing with an emphasis on abdominal wall movement and diaphragmatic action, in addition to the seated position during the protocol, which also optimizes the stress-length relation of the diaphragm. In this context, the X5 parameter, which reflects the elastic recoil of peripheral airways, also improved after DBE, suggesting that respiratory control has an impact on more distal regions of the bronchial tree.^24^ However, despite the decrease in these oscillometric parameters after DBE, they did not return equally to baseline values, prompting further investigation with a larger number of technique repetitions.

To the best of our knowledge, DBE can be applied as an important therapeutic strategy in individuals with respiratory mechanics abnormalities, such as those with CF.^6^ Positive outcomes were found in a diaphragmatic breathing training program in individuals with chronic obstructive disease, who exhibited improved functional capacity and increased diaphragmatic mobility after four weeks of supervised sessions with a single physiotherapist.^21^ The program consisted of 12 sessions, during which patients performed three sets of ten BDE repetitions in different positions (supine, right, and left lateral decubitus, sitting, and standing). Throughout the sessions, patients received tactile feedback on their abdominal and upper chest, in addition to visual and auditory cues as needed.

One limitation of the present study was the absence of individuals with more severe disease, which might have affected the analysis of different outcomes in response to the intervention protocol, as well as other possible groupings according to CF manifestations. In summary, this study investigated the effects of each FET stage on the respiratory mechanics of schoolchildren with CF. The findings revealed an immediate increase in airway resistance after a sequence of five low-volume huffs followed by three high-volume huffs. The stage consisting of ten DBE repetitions resulted in a reduction of oscillometric parameters, which had previously increased following the huff sequence, emphasizing the importance of incorporating relaxation periods during this intervention to promote airway stabilization in children with CF. However, the ten DBE repetitions were not sufficient for the oscillometric parameters to return to baseline values, highlighting the need for future studies with a greater number of DBE repetitions.

It is important to highlight that, during the execution of this protocol, no patient experienced destabilization due to increased airway resistance. However, in light of these findings, it is essential to discourage the indiscriminate use of huffing in children with CF, ensuring that it is performed based on the recommendations made by a physiotherapist. Furthermore, the importance of practicing DBE is emphasized, along with the need to encourage new studies with a greater number of repetitions of this technique.

The occurrence of coughing episodes in some participants during the protocol appears to be associated with a more obstructive pre-existing condition.

In conclusion, applying the FET protocol in children and adolescents with CF resulted in increased airway resistance immediately after the huff sequence. The DBE reduced the increased resistance values caused by the huff sequence; however, ten repetitions of DBE were not sufficient to restore the oscillometric values to baseline levels immediately after the huff sequence. Coughing did not significantly impact any of the parameters studied.

## Data Availability

The database that originated the article is available with the corresponding author.
